# Long‐term therapeutic effect in nonhuman primate eye from a single injection of anti‐VEGF controlled release hydrogel

**DOI:** 10.1002/btm2.10128

**Published:** 2019-06-10

**Authors:** Yu Yu, Xingyan Lin, Qilin Wang, Mingguang He, Ying Chau

**Affiliations:** ^1^ Department of Chemical and Biological Engineering The Hong Kong University of Science and Technology Kowloon Hong Kong, China; ^2^ State Key Laboratory of Ophthalmology, Zhongshan Ophthalmic Center Sun Yat‐sen University Guangzhou China; ^3^ The Hong Kong University of Science and Technology Shenzhen Research Institute Shenzhen China

**Keywords:** anti‐VEGF, controlled release, drug delivery, hydrogel

## Abstract

Wet age‐related macular degeneration (wet‐AMD) is a leading cause of irreversible blindness. Current treatment of AMD requires monthly intravitreal injection, which is difficult to be implemented in many parts of the world. In recent years, controlled release of anti‐vascular endothelial growth factor (VEGF) therapeutics has attracted intense research interest aiming to reduce the injection frequency to one or two times per year. In this study, we evaluated the in vivo pharmacokinetics and the long‐term therapeutic efficacy of an in situ hydrogel encapsulating an anti‐VEGF antibody in nonhuman primates. We show that after a single injection of anti‐VEGF controlled release hydrogel, a relatively constant concentration of drug can be maintained in the monkey eye for at least 5 months and the dose was sufficient for the treatment of recurrent choroidal neovascularization induced by repeat laser photocoagulation in monkeys. Our result suggested that when formulated into a controlled release formulation, a single dose of anti‐VEGF may be sufficient for a half‐year treatment and controlled release may be a suitable strategy to reduce the injection frequency in the treatment of AMD in human.

## 1 INTRODUCTION

Wet age‐related macular degeneration (wet‐AMD) is one of the leading causes of irreversible blindness.^1^, ^2^, ^3^ Intravitreal anti‐vascular endothelial growth factor (VEGF) therapy has been the first line treatment for wet‐AMD since the introduction of anti‐VEGF antibody/antibody fragment (bevacizumab or Avastin [off label use for ophthalmic indications], and ranibizumab or Lucentis) in 2005.^4^, ^5^ Compared to pre‐anti‐VEGF treatment options, including vitamin supplies and photodynamic therapy, where the patient's visual acuity (VA) would continue to decline after treatment,^6^, ^7^ intravitreal injection of anti‐VEGF is able to improve patients' VA. However, long‐term and frequent injection is required in order to achieve continuous VA improvement.^8^ Several retrospective analyses have demonstrated that at least about 10 injections per year is required if the therapeutic benefit is to be sustained over multiple years.^9^, ^10^ Switching to a lower injection frequency was associated with suboptimal treatment and eventually led to a decline in VA.^11^, ^12^, ^13^

Monthly injection creates huge inconvenience and cost to the patients, hospitals, and societies. The issue is beyond patient compliance. It is practically impossible, in terms of the requirement of financial and healthcare resources, for most patients to maintain such injection frequency.^14^ Similarly, anti‐VEGF is also effective in treating diabetic macular edema and proliferative diabetic retinopathy, but the requirement of repeated injection forced most patients to choose the less effective laser treatment.^15^

To overcome these obstacles, intravitreal controlled release systems that aim to prolong the therapeutic duration of anti‐VEGF has been actively pursued over the past 10 years in both industry and academia. However, the following problems have been encountered: (a) the time for protein release is not long enough; (b) the depot fails to preserve the protein activity; (c) the delivery vehicles are not compatible with ocular tissues.^16^, ^17^, ^18^, ^19^, ^20^ The lack of an animal model to evaluate the prolong therapeutic duration of these formulations brings an additional barrier to the translation of these devices to clinical use.^18^

Previously, we developed an injectable hydrogel composed of hyaluronic acid grafted with vinylsulfone (HA‐VS) and dextran grafted with thiol (Dex‐SH). Using the Blob model as a guidance for rational design, we were able to identify formulations that released bevacizumab in vivo in rabbit eyes for over 6 months with excellent biocompatibility.^21^, ^22^ In this study, we aim to confirm the ocular biocompatibility in nonhuman primates, and evaluate the in vivo pharmacokinetics and long‐term therapeutic effect of the controlled release formulation in a nonhuman primate model with recurrent choroidal neovascularization (CNV).

## 2 MATERIALS AND METHODS

### 2.1 Animals

Male rhesus monkeys (Guangdong Landau Biotechnology Co., Ltd. Guangzhou, China) aged 2–4 years, weighting 3–5 kg, were used in this study. All animal experiments adhered to the guidelines of the Association for Research in Vision and Ophthalmology Statement for the Use of Animals in Ophthalmic and Vision Research and were approved by the animal ethics committee of Zhongshan Ophthalmic Center at Sun Yat‐sen University (Acceptance number 2017–020). For all ophthalmic procedures including laser photocoagulations, fundus imaging, ERG, and intraocular pressure measurement, the animals were anesthetized with intramuscular injection of 30 mg/kg pentobarbital followed by pupillary dilation with tropicamide and topical application of Alcaine on the ocular surface. For ethical reasons, the same monkey was used for biocompatibility, in vivo pharmacokinetics as well as the therapeutic effect evaluation (including CNV model construction).

### 2.2 Preparation of chemically crosslinkable polymers

HA‐VS and Dex‐SH were prepared according to our previous publications.^21^, ^22^ Briefly, HA‐VS was made by reacting 1.25x (DVS:OH) molar excess of DVS with HA in 0.1 M NaOH solution and stopped by neutralizing with HCl. Dextran was first converted to Dex‐VS by reacting with 1.25x (DVS:OH) molar excess of DVS in 0.02 M NaOH. Dex‐SH was made by reacting Dex‐VS with excess amount of DTT in N_2_. The polymers were purified by tangential flow filtration (mPES MidiKros TFF Filter, NMWL = 3 kDa, D02‐E003‐05‐N, Spectrum Laboratory Inc.), adjusted to pH 5.2, sterile filtered with 0.22 um syringe filter (Millex GP, Merk Millipore Ltd., Cork, Ireland) and freeze‐dried. The characterization of polymers was performed according to our previous publications using ^1^H NMR and Ellman's assay.^21^, ^22^, ^39^ The degree of modification (DM) for HA‐VS and HA‐SH used in this study was similar to the previous study.^22^

### 2.3 Retinal laser photocoagulation

Photocoagulation was performed unilaterally on the same eye for two times at predetermined time points. At each time, four burns were usually created aligning with macula at one to three optic disc diameter away from the fovea. The number of laser burns may be reduced to two or three when deemed necessary to avoid damaging the large choroidal vessels. The large choroidal vessels were identified by imaging the eyes with infrared mode or automatic real time mode of a confocal scanning laser ophthalmoscope (Spectralis HRA + OCT, Heidelberg Engineering, Germany) before laser treatment. Spectral domain optical coherence tomography (SD‐OCT, Spectralis HRA + OCT, Heidelberg Engineering, Germany) was used to confirm the laser location and Bruch's membrane (BM) rupture. The initial laser power was set as 700 mW for the production of the vapor bubbles. The spot size and the duration of the laser were fixed at 50 μm and 0.1 s, respectively. A successful laser spot was defined as the rupture of BM. If the first burn failed to break the BM as determined by SD‐OCT, an additional shot was delivered with an incremental power of 200 mW till the BM was confirmed to be ruptured.

**SCHEME 1:**
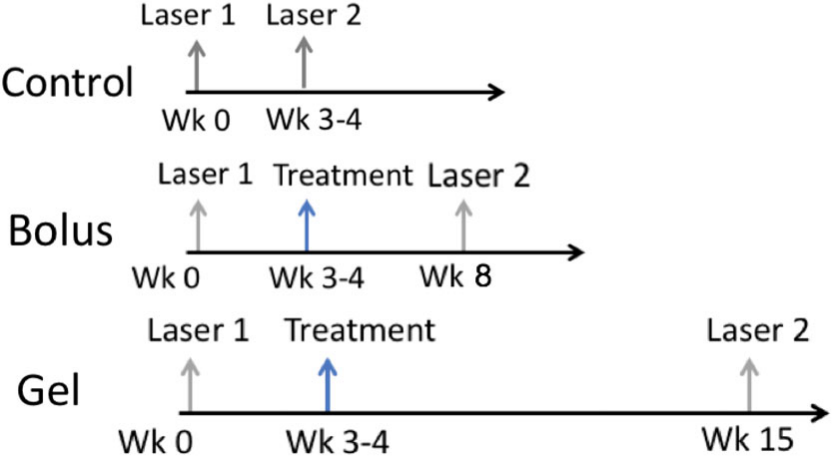
Laser and drug injection schedule in the current study

The animals were divided into three groups of *n* ≥ 3 (Scheme 1). For the control group, the second laser photocoagulation (Laser 2) was performed 4 weeks after the first laser photocoagulation (Laser 1). For both bevacizumab bolus injection (bolus group) and hydrogel formulation group (gel group), the drug was injected to the laser‐treated eyes 3 to 4 weeks after Laser 1. For the bolus group, Laser 2 was performed 4 weeks after drug injection. For gel group, Laser 2 was performed 12 weeks after gel injection when the aqueous concentration was similar to that at Week 4 post bolus injection.

### 2.4 ffERG measurement

The full‐field electroretinography (ffERG) test was conducted before laser induction and after gel injection using an electrophysiological unit (Reti‐port, Roland, Germany). The procedures, applied before the laser treatment and 3 days after the injection, were based on the ISCEV standard with six basic responses.^40^ Briefly, the ground electrodes were subcutaneously inserted into the forehead skin and the reference electrodes were put into the lateral side skin of both eyes. Then the JET contact electrodes were placed on the cornea. The impedance was checked to make sure that the value was <10 kΩ by adjusting the electrode position. After at least 20 min of dark adaption, scotopic response 1 to 4 were recorded sequentially. After 10 min of light adaption, photopic response 5 to 6 were also recorded on both eyes.

### 2.5 Fundus photography

The monkey's fundus was imaged by Pictor Plus (Volk Optical Inc., OH). The illumination level was set to 1. The head was at the upright position similar to human fundus imaging. To better image the hydrogel, after hydrogel injection an InView lens (Volk Optical Inc., OH) adapted with an iPhone 6 was use. The monkeys were laid down with eyes facing up for InView imaging. The InView can better image the gel because of the larger visual field, thicker focal plane and the hydrogel in some cases will move toward the imaging field when the monkeys eyes were facing up.

### 2.6 Fundus fluorescein angiography and CNV scoring

Fundus fluorescein angiography was performed with a Pictor Plus adapted with a fluorescein angiography module (Volk Optical Inc., OH). The illumination level was set to 3. The monkeys were quickly perfused with 10% fluorescein (Guangzhou Baiyunshan Pharmaceutical Co. Ltd., Guangzhou, China) through the vein at a dose of 0.05 mL/kg body weight. Fundus angiograms were obtained in the early (∼10 s after injection) and late phase (∼ 6 min). The late‐phase angiogram was compared to the corresponding early phase image for CNV scoring. A I to IV classification system according to a previous study was used^24^, in which I = no hyperfluorescence; II = hyperfluorescence without leakage; III = hyperfluorescence early or mid‐transit with late leakage; IV = hyperfluorescence early or mid‐transit with late leakage extending beyond the borders of the treated area.

### 2.7 Percentage of CNV spot remaining after treatments

For each treatment group, the total number of Grade III or IV CNV spots of the three monkeys were counted at different time point. The percentage of CNV spot remaining is calculated from the total number of Grade III or IV CNV spots at the time of measurement divided by the maximum number of Grade III or IV CNV spots developed. The remaining % of Grade III or IV CNV spot were plotted similar to a survival plot. Logrank test for trend were performed using Prism 7 to calculate the *p* value.

### 2.8 Intravitreal injection of bolus or gel‐formulated bevacizumab in monkey eyes

Bevacizumab (bolus or gel formulation) was injected to the monkey eyes after CNV leakage (Grade III/IV) were fully developed in the monkey eyes (usually about 3–4 weeks). Before the injection, the ocular surface was sterilized by tobramycin eye drops (Hangzhou Guoguang Pharmacuetical Limited, Hanghzou, China). For gel formulation, Avastin solution (25 mg/mL, Avastin Roche Diagnostics GmbH, Germany) was first adjusted to pH 7.0 by dropwise addition of 1 M NaOH. This solution was used to dissolve HA‐VS and Dex‐SH. The two polymer solutions were cooled on ice. Afterwards, the polymers were mixed, loaded into a precooled syringe, and injected to the right eye of the monkey via a 31‐gauge needle at the pars plana 2 mm behind the limbus at the superior temporal region toward the direction of the optic disk. The final formulation of the gel was composed of 25 mg/mL bevacizumab, 12% (wt/vol) 29 kDa HA‐VS of 15% DM, and 24% (wt/vol) 40 kDa Dex‐SH of 13% DM. The volume for each injection was about 50 μL which contained 1.25 mg bevacizumab. For bolus bevacizumab injection, 50 μL (1.25 mg) Avastin solution was injected at a location similar to gel injection. After injection, the needle stayed in the vitreous cavity for 10 s before withdrawal to avoid the backflow. Tobramycin ointment was applied on the eye surface to avoid infection.

### 2.9 Enzyme‐linked immunosorbent assay

The concentration of bevacizumab in the aqueous humor was measured by Sandwich Enzyme‐linked immunosorbent assay (ELISA). Aqueous humor samples of about 175 μL were taken out from the anterior chamber using a 1 mL syringe attached to a 28G needle. The samples were immediately placed on ice and taken to the laboratory for further processing. Bovine serum albumin (BSA, 10%) (MP Biomedicals New Zealand Limited, Auckland, New Zealand) of 1/10 volume of the aqueous sample was added to each sample. ELISA was performed either on the day of sample retrieval or 1 day after (stored in −80°C freezer before examination), and a standard curve (*n* = 3) was made each time when the test was performed. To perform the ELISA, 90 μL of 0.125 μg/mL VEGF (SinoBiologics, Beijing, China) was coated on the high affinity 96‐well plate (JET BIOFIL, China) for overnight at room temperature. After blocking with 350 μL of 1% BSA dissolved in PBS, 100 μL samples or standards (bevacizumab solution of known concentrations) were incubated for 2 hr, followed by 1 hr of incubation with 100 μL of 33 ng/mL anti‐human IgG antibody conjugated with HRP (SinoBiologics, Beijing, China). Afterwards, 100 μL one‐component tetramethylbenzidine‐based substrate (Beyotime, Guangzhou, China) was added and the reaction was stopped by adding 50 μL of 2 M HCl. Between each step, each well was washed with 300 μL of washing buffer (PBS +0.05% Tween‐20) for three times. The linear range of bevacizumab concentrations using this method is from ∼300 pg/mL to ∼14 ng/mL. The number of monkeys and eyes for the measurement of pharmacokinetics was three eyes of three monkeys for bolus bevacizumab and five eyes of four monkeys for gel formulation. The reason for having two more eyes for the gel formulation is because we performed a preliminary pharmacokinetics experiment on one monkey by injecting the gel to both eyes. The purpose of the experiment is to evaluate if the time interval we chose for the monkey study (based on our pervious rabbit study) is suitable. The results of the two eyes were combined with the formal study.

### 2.10 Inflammatory cytokine measurement

Inflammatory cytokines in the aqueous were measured by MILLIPLEX® Multiplex Assays (Non‐Human Primate Cytokine/Chemokine Panel 1, Merk). The assay was performed by Asbio Technology Inc. (Guangzhou, China) using Magipix system (Luminex Corporation) according to the manufacturer's instruction. The aqueous samples were stored in 1% BSA and frozen at −80°C before performing the assay. For each time points, three animals were used and duplicates were performed for each animal. The measured intensity was compared to standard curves per manufacturer's instruction. The cytokine tested were G‐CSF, GM‐CSF, IFNγ, IL‐1β, IL‐1ra, IL‐2, IL‐4, IL‐5, IL‐6, IL‐8, IL‐10, IL‐12/23(p40), IL‐13, IL‐15, IL‐17A, MCP‐1, MIP‐1β, MIP‐1α, sD40L, TGF‐α, TNF‐α, VEGF, and IL‐18.

## 3 RESULTS

### 3.1 Ocular biocompatibility in monkeys

**Figure 1:**
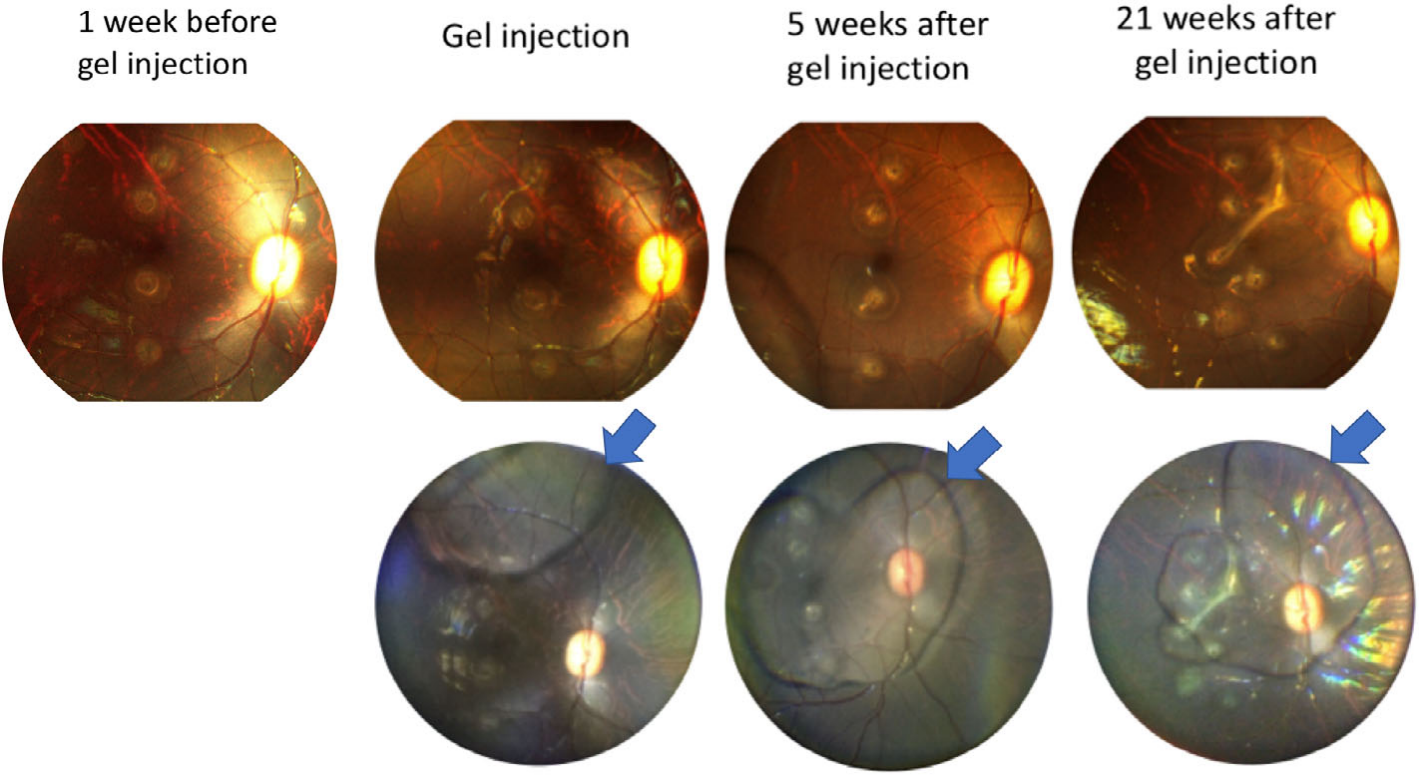
Fundus images of a monkey eye before and after intravitreal injection of bevacizumab‐encapsulating gel. Upper panel: Pictor Plus images. Lower panel: InView images. The images of the upper and lower panel were of the same eye. The locations of the gel were indicated by arrows

Fundus imaging was performed daily for the first 3 days post hydrogel formulation injection, weekly in the first month, and biweekly in the second and third month, and once in the 21st week. No gross changes in the monkey retina, including hemorrhage, retinal detachment, edema, or vitreous haze was observed by either Pictor Plus or InView. The hydrogel appeared transparent under InView. Representative fundus images are shown in Figure 1.

**Figure 2:**
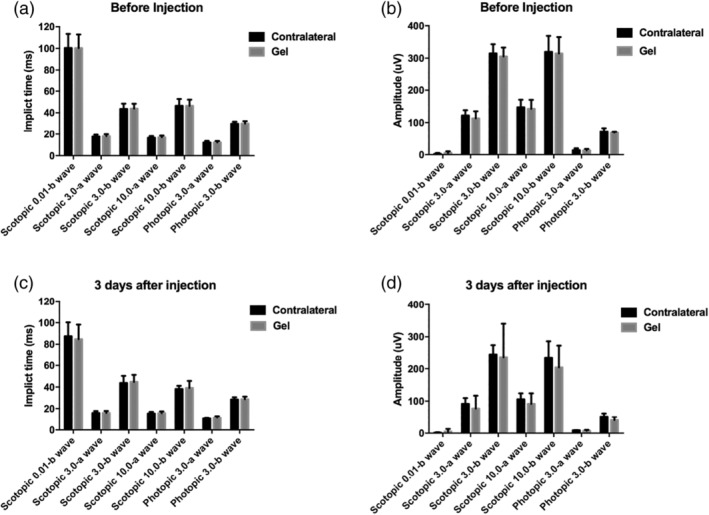
Comparison of retinal activities (implicit time and amplitude) by ffERGs before (a, b) and after (c, d) intravitreal injection of bevacizumab encapsulated gel. The data were represented as mean ± *SD*

Full‐field electroretinography (ffERG) was measured for both eyes before any treatment and 3 days after gel injection. ffERG is a tool to evaluate the sum of the activity of all retinal cells: scotopic 0.01 b‐wave reflects the activity of rods, scotopic 3.0 a‐wave and b‐wave reflect the activity of photoreceptors and bipolar cells, scotopic 10.0 a‐wave and b‐wave reflect the activity of postreceptoral and predominantly rod bipolar cells, scotopic 3.0 osciliatory potential response reflects the activity of middle retinal layer and the function of vascular, and photopic 3.0 a‐wave and b‐wave reflect the activity of cone cells. In each response, there were no significant differences of implicit time (Figure 2a,c) and amplitude (Figure 2b,d) between the gel injection eye and contralateral eye, indicating that the gross neuronal activities of retinal cells were normal after gel injection.

**Figure 3:**
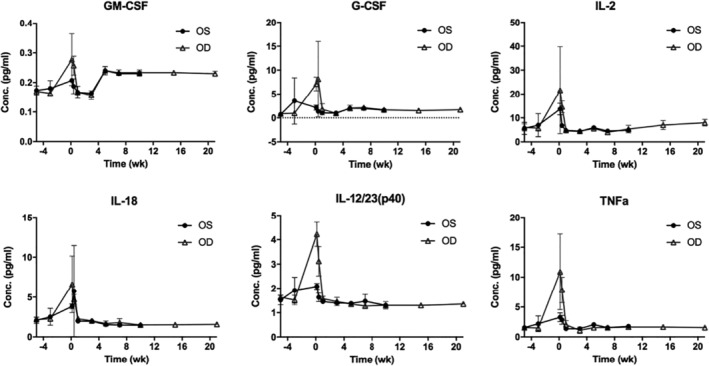
The change of inflammatory cytokines concentration before and after gel injection. OS: contralateral eye. OD: gel injection eye. The gel injection day was denoted as Week 0 at the X axis. Five and three weeks before gel injection (Week −5 and −3 at the X axis), the baseline level of cytokines were measured. The data were represented as mean ± *SD*

All inflammatory cytokines tested, including proinflammatory and anti‐inflammatory cytokines (e.g., IL‐10), showed an increase in concentration after gel injection but quickly returned to baseline within the first week. Representative examples are shown in Figure 3. The complete set of analysis is shown in Supporting Information Figure S1.

### 3.2 In vivo pharmacokinetics in monkey eyes

**Figure 4:**
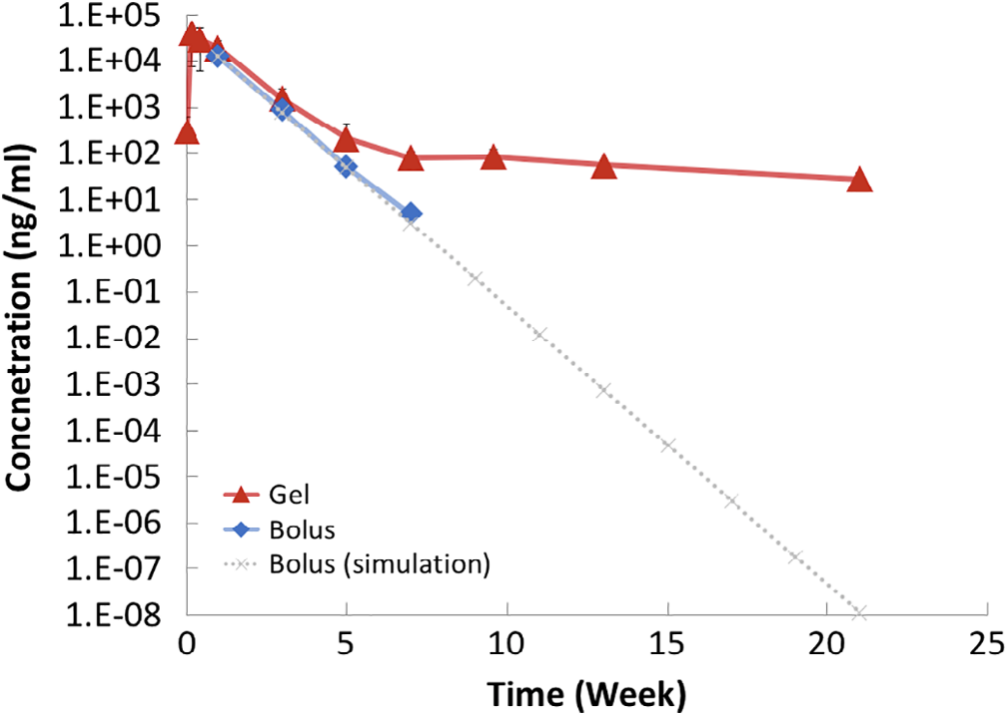
Aqueous pharmacokinetics of bevacizumab following intravitreal injection of bolus or gel formulation. The last measurable time point for bolus injection was in the 7th week (5 ± 3.5 ng/mL), the dotted line represents the simulated concentration assuming first order elimination kinetics. The data are represented as mean ± *SD*

The aqueous pharmacokinetics of intravitreally injected bolus bevacizumab in monkey eye showed an exponential decay similar to the results in previous studies (Figure 4).^18^, ^23^ The aqueous elimination half‐life calculated assuming first order elimination was 3.5 days, which is similar to the value calculated by Miyake et al. (2.8 days).^16^ The in vitro release kinetics and the in vivo rabbit eye pharmacokinetics of the present hydrogel has been described previously. The hydrogel was able to release bevacizumab at therapeutically relevant level for at least half year in vivo in rabbits eyes.^21^, ^22^ In the present monkey study, we found that for the first 4 weeks, the pharmacokinetic profile of the gel group matched closely to that of the bolus group, although the concentration was slightly higher. Afterwards, a much slower decay with a half‐life of about 3 months was observed. Thus, the gel group showed a different pattern of concentration‐time profile in the aqueous humor when compared to the bolus group. The concentration of bevacizumab in the aqueous was 78 ± 27 and 27 ± 10 ng/mL (mean ± *SD*) in Week 7 and Week 21 post gel injection; whereas the value in Week 4 was 50 ± 32 ng/mL (mean ± *SD*) after bolus injection.

### 3.3 Antiangiogenic effect against recurrent laser induced CNV

**Figure 5:**
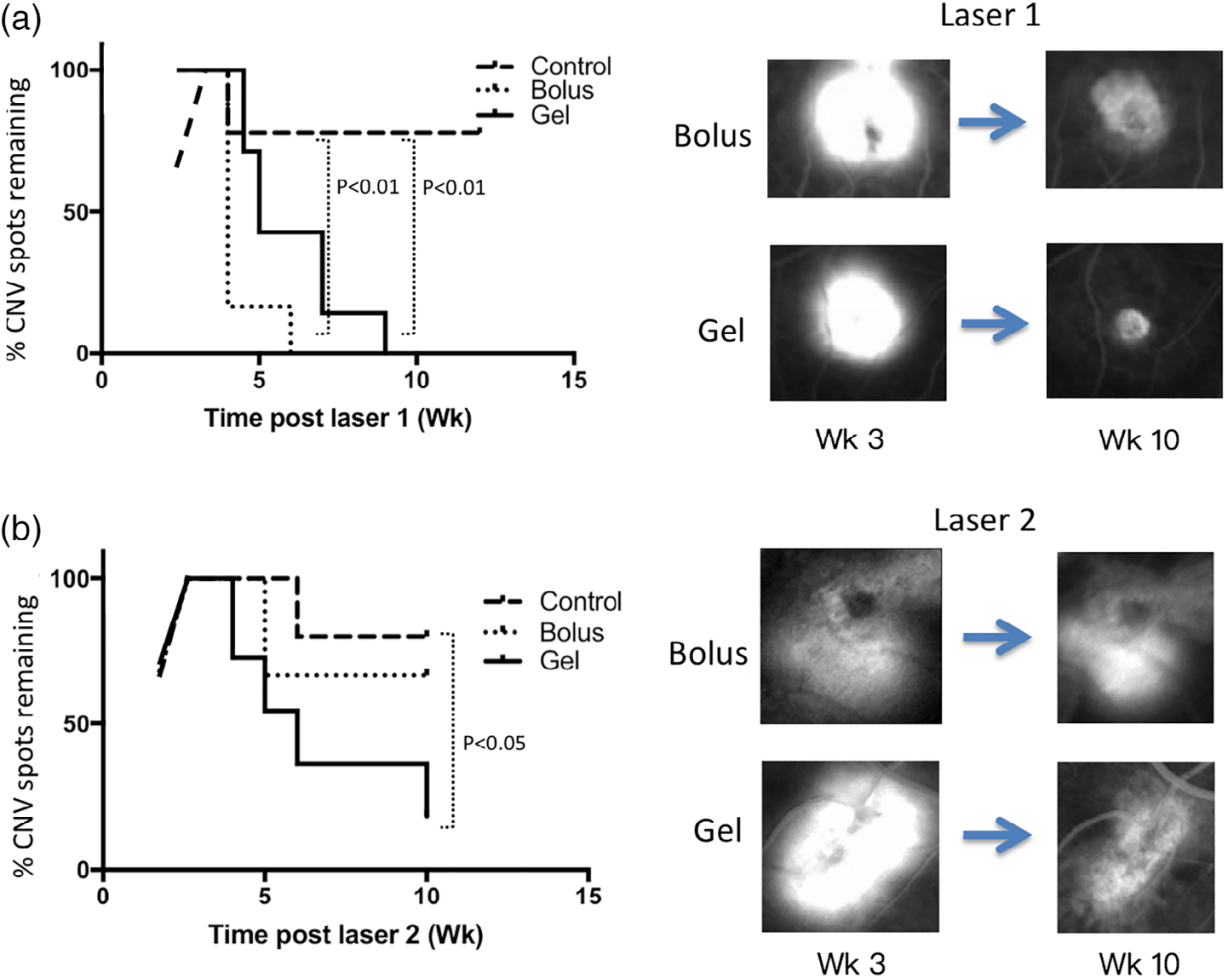
(a) The change of percentage of CNV spots remaining after the peak level post Laser 1 induction. (b) The change of percentage of new CNV spots remaining after the peak level post Laser 2 induction. Bolus groups were monkeys injected with a bolus injection of Avastin. Gel groups were monkeys injected with bevacizumab encapsulated hydrogel. Control groups were monkeys treated with laser photocoagulation only. For each group *n* ≥ 3. The *p* value refers to comparison between control group and the two treatment groups for (a), and control group with gel injection group for (b)

At least one CNV spots of Grade III or IV was successfully induced in each monkey after each laser induction. This consistency has enabled the evaluation of long‐term effect of anti‐VEGF releasing depot and the comparison of different treatment groups with small number of monkeys. The therapeutic effect of bolus and hydrogel treatments were evaluated by following the recovery of CNV spots to lower grades. The laser induction and injection schedule are shown in Scheme 1. The total number of Grade III or IV CNV spots of the three monkeys were counted for each group at different time point. The percentage of CNV spot indicated the number of CNV spots at the time of measurement over the maximum number of CNV spots developed during the examination. Figure 5 shows the change of percentage of CNV spots remaining after the peak level reached post two laser inductions.

After each laser treatment, it took 3–4 weeks for the CNV spots to develop and reach the maximum value. In the control group receiving no bevacizumab, 80% of the CNV spots remained Grade III or IV for at least 3 months (Figure 5). In the group receiving bolus injection, the CNV spots induced by Laser 1 vanished entirely after the treatment, confirming the expected therapeutic effect of bevacizumab^24^, ^25^ (Figure 5a). Subsequent to the complete resolution of the CNV spots by Laser 1, Laser 2 was performed for this group in Week 8 (Scheme 1), which was 4 weeks after bolus injection. However, the low and exponentially decreasing concentration of bevacizumab neither prevent nor resolve the CNV spots induced by Laser 2 (Figure 5b). The percentage of CNV spots followed the same trend as control group, with an initial modest decrease (considered as self‐recovery) in the first month and remained the same for the rest of the study. The number is slightly lower than the control group (67% vs. 80%).

In the gel group, the CNV spots induced by Laser 1 also vanished entirely albeit at a slightly slower rate compared to the bolus group (Figure 5a). Laser 2 was performed on this group in Week 15 (Scheme 1). The time of Laser 2 was chosen when the aqueous bevacizumab concentration in the gel group was similar to that of the bolus group at the time of its Laser 2 induction. Both groups exhibited a value about 50 ng/mL. Post Laser 2, the percentage of CNV spots in the gel group decreased immediately after its full development (4 weeks post laser), and continued to decrease over 10 weeks to 18%, which is significantly lower than the control group (*p* < 0.05, Figure 5b). Therefore, antiangiogenic effect was observed for the gel formulation after recurrent CNV. The therapeutic effect was extended to at least 5 months.

## 4 DISCUSSION

Wet AMD and DR are serious public health problems because it could lead to blindness if not treated properly.^26^, ^27^ Posterior eye diseases are the leading causes of blindness in developed countries.^28^ The invention of anti‐VEGF therapy and the success of clinical use were encouraging; however, the once‐per‐month injection scheme for the management of chronic eye diseases was impossible to be implemented in most parts of the world. In recent years, there has been a growing interest to reduce the number of anti‐VEGF injection. It was postulated that VEGF could be a neurotrophic factor and monthly frequent injection might cause retina and retinal pigment epithelium damage, causing geographic atrophy and in the long run deteriorate patients' vision.^29^ However, new evidence has shown that prolonged VEGF blockage is not neurotoxic and monthly injection of anti‐VEGF is necessary to maintain a patient's long‐term visual gain.^11^, ^30^, ^31^

In order to reduce the injection frequency, gene therapy and controlled release devices are the two major areas of research. Gene therapy has the potential to ultimately reduce injection to one time only. However, recent clinical trials suggested that the control of viral infection and expression is far from optimum.^32^ Controlled release device on the other hand offers a potentially simpler solution that can be translated to clinics in a shorter time because the antibody drug has already been proven effective and safe. Various types of devices, including micropumps, polymeric reservoirs, polymeric implants, polymeric particles, hydrogels, and hybrid systems, have been developed in recent years.^18^ However, despite the intense efforts in the past decade, only two implant devices have been evaluated in early phase clinical trials and the results were unsatisfactory.^18^ The major issues for formulating anti‐VEGF are related to ocular biocompatibility, release kinetics, and protein activity. In this study, we have demonstrated that the hydrogel depot of anti‐VEGF protein we developed has the potential to overcome these issues.

For biocompatibility, we previously demonstrated that our polysaccharide based, chemically crosslinked in situ hydrogel was well tolerated in rabbit eyes.^22^ HA was chosen as one of the main components because it is native to the eye.^33^ Both HA and dextran have been used extensively in ophthalmological applications and have excellent safety records.^34^ However, controlled release devices that are well tolerated in rabbits may still elicit inflammation and other incompatibility problems in primates.^16^ To address this question, the hydrogel encapsulating bevacizumab was injected to monkey eyes intravitreally to evaluate short‐term and long‐term biocompatibility. The retinal function was tracked by electroretinography (ERG). The wave form, implicit time, and amplitude of the retinogram were analyzed. The result showed that there was no statistical difference between the gel‐injected eye and the contralateral eye, as well as for the same eye before and after gel injection (Figure 2). The monkeys in the gel group could also responded well to the visual stimulation provided by the investigators. The morphology of the retina was evaluated by fundus imaging. We found that the presence of hydrogel did not induce gross changes in the retina. No hemorrhage, retinal detachment, edema, or vitreous haze was found (Figure 1). Furthermore, the vitreous and hydrogel were found to be clear and transparent throughout the study, indicating a lack of inflammatory response. To further confirm this finding, we measured 24 representative inflammatory cytokines in the aqueous humor before and after injection. The aqueous concentration of inflammatory cytokines has been shown to be well‐correlated to the vitreous concentration and pathological states.^35^, ^36^, ^37^, ^38^ The results showed that a spike of upregulation of all cytokines were observed after injection, but the level quickly returned to the baseline within a week and stayed the same throughout the study (5 months) (Figure 3 and Supporting Information Figure S1). This result suggested that the cytokine spike may be a result from the injection and the presence of gel did not induce chronic inflammatory response.

We showed that the hydrogel was able to release bevacizumab in monkey eyes for over 5 months (Figure 4). In Week 21, the measured concentration of bevacizumab in the gel group was 27 ± 10 ng/mL, which is much higher than the expected concentration for bolus injection (∼10^−8^ ng/mL) and same in magnitude as that in the bolus group in Week 4 post injection (50 ± 32 ng/mL). The amount of drug and the volume injected was identical for the bolus and the gel group. To evaluate if the continuous slow release of bevacizumab is therapeutically effective, a recurrent monkey CNV model by repeated laser induction was used. In this model, a small number of laser burns (∼2–4) were first photocoagulated in the monkey eye. After the development of CNV, drugs were injected to the monkey eyes to evaluate the antiangiogenic effect. Afterwards, the same monkey eye was photocoagulated again at the predetermined time points, and thus the therapeutic duration of the drug can continue to be evaluated. Compared to other models in which the induction needs to be performed on different monkeys, this method eliminates the potential individual difference between monkeys^18^ and thus significantly reduces the number of monkeys required. Our results demonstrated that the hydrogel formulation was able to resolve the CNVs induced by Laser 1 similar to the bolus injection (Figure 5). This happened within 2–3 weeks after injection. This result correlates well with the pharmacokinetics data (Figure 4) where the concentration of bevacizumab is similar between the group receiving bolus and the group receiving gel injection in the first month. Post Laser 2, the bolus group was similar to the control group. The CNV development peaked at about 3 weeks after laser photocoagulation, with about 20% of CNV resolved spontaneously in about a month and the majority of CNV spots remained for at least another 2 months. There is no statistical significant between the control group and the Avastin group. In contrast, in the group injected with gel formulation, CNV was developed but resolved gradually. For the last time point, 83% of CNV spots were resolved (*p* < 0.0.5 compares to control group). Although the statistical analysis is less meaningful because of the small number of animal and the large variation of the monkey, our results indicate that: (a) the protein encapsulated in the hydrogel is likely to be still active after 3–5 months; (b) the concentration of active drug in the vitreous is likely to be therapeutically effective when maintained for a prolong period of time. In this study, we followed the percentage change of total number of Grade III and IV CNV spots for each group. In essence, this analysis showed the drying of the leakages from newly grew vessel which we believe will better reflect the therapeutic effect. The change of total fluorescent intensity of Grade III and IV CNV spots for each group were also summarized in Supporting Information Figure S2 as a reference. It should be noted that, assuming the hydrogel does not accelerate the clearance of the drug in the eye, the bevacizumab concentration measured in the aqueous would be about 5 times lower than vitreous concentration for human,^18^ and the clearance rate in monkey would be about 2 times faster than human.^18^ Thus the hydrogel controlled release system warrants further effort for translation into clinical use.

## AUTHOR CONTRIBUTIONS

Y.Y. contributed experimental design, experiment execution, data analysis, and manuscript preparation; X.L. contributed experiment execution and data analysis; Q.W. contributed experiment execution; M.H. contributed data analysis; Y.C. contributed experimental design, data analysis and manuscript preparation; Y. Y. and Y. C. are shareholders of Pleryon Therapeutics Ltd.

## Supporting information

Fig. S1. The change of inflammatory cytokines concentration before and after gel injection. OS: contralateral eye. OD: gel injection eye.
Fig. S1. The change of MIFI value after different treatment
