# Supplementary material for: Long‐term therapeutic effect in nonhuman primate eye from a single injection of anti‐VEGF controlled release hydrogel
Source: Bioeng Transl Med. 2019 Jun 10;4(2):e10128. doi: 10.1002/btm2.10128 (PMC6584386; doi:10.1002/btm2.10128)
Supplement: Supplementary file 1 — Fig. S1. The change of inflammatory cytokines concentration before and after gel injection. OS: contralateral eye. OD: gel injection eye. Fig. S1. The change of MIFI value after different treatment [file BTM2-4-na-s001.docx]

**Supplementary Materials**

Fig. S1. The change of inflammatory cytokines concentration before and after gel injection. OS: contralateral eye. OD: gel injection eye.


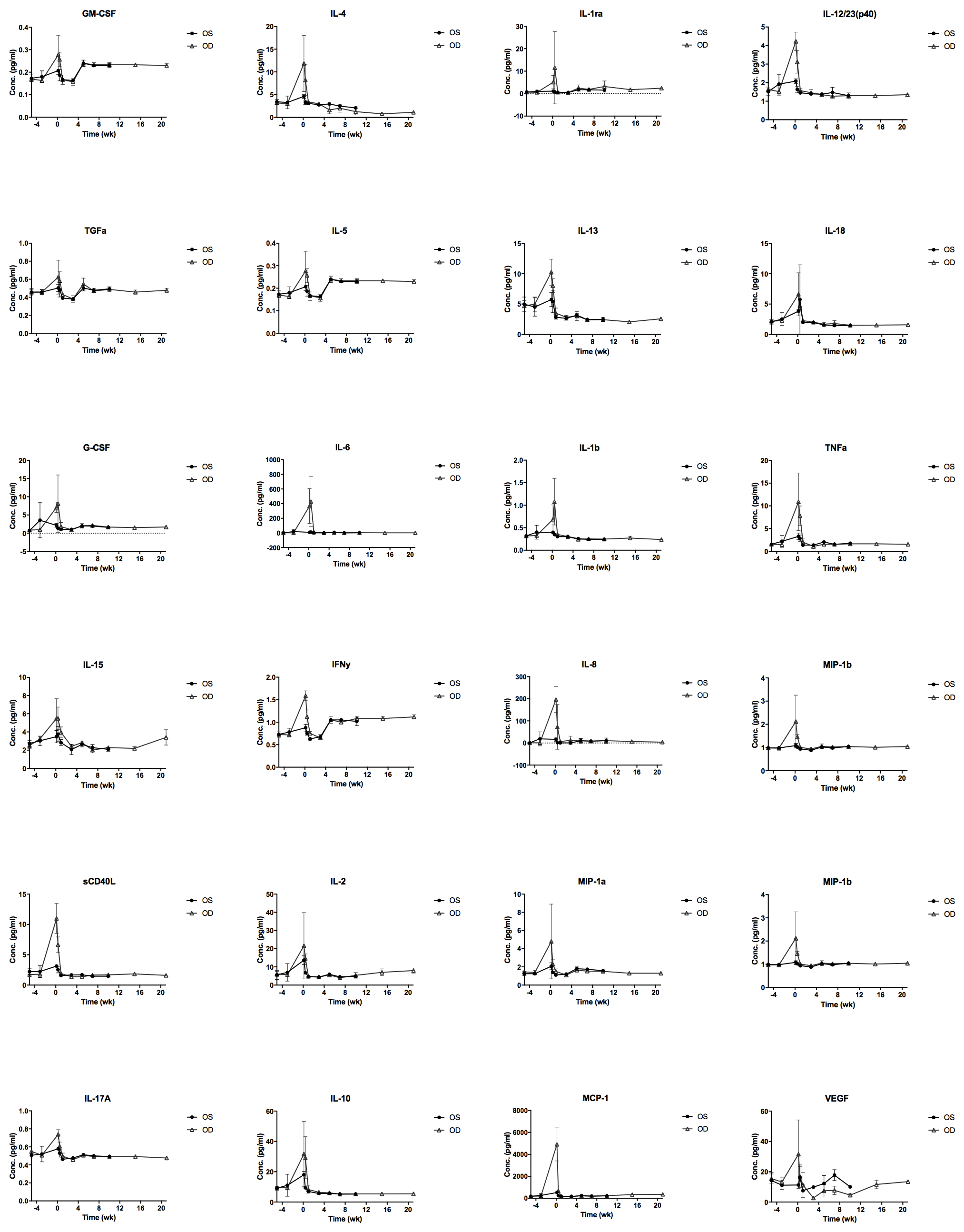


Fig. S1. The change of MIFI value after different treatment


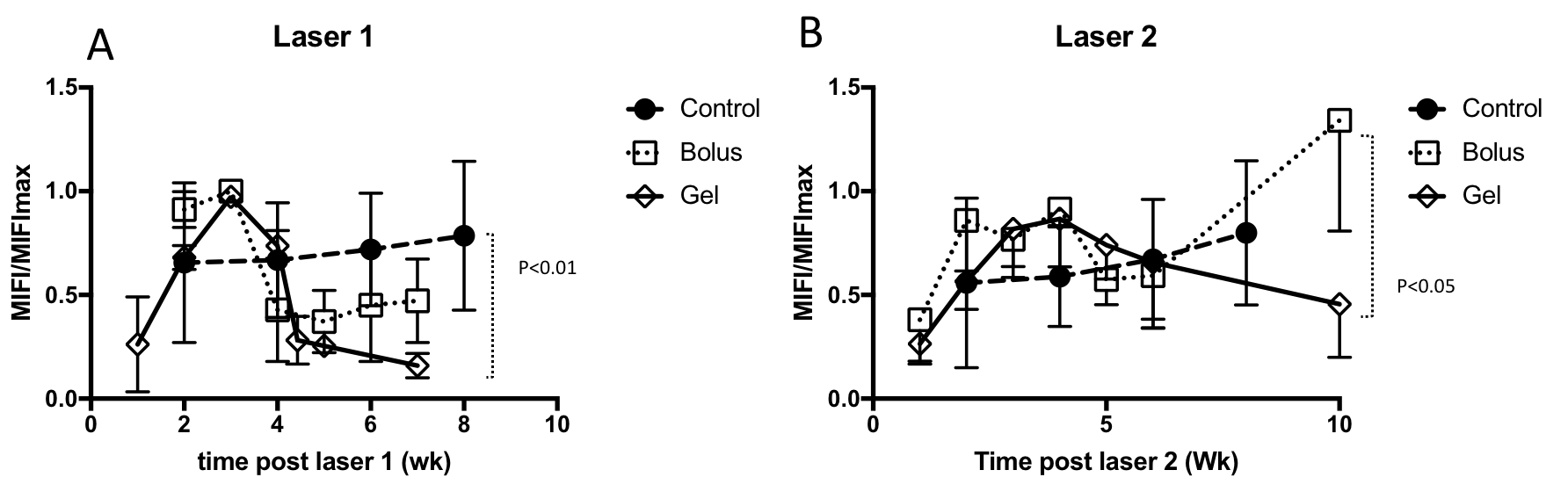


The late phase angiograms of each eye at each time points were analyzed with the Image-Pro Plus software (Image-Pro Plus 6.0, Media Cybernetics, Inc) and the mean integrated fluorescence intensity (MIFI) of lesions created in each laser was calculated as the following:

$MIFI={(CLOD1\times Area1+CLOD2\times Area2+\ldots+CLODn\times Arean)}/{N,}$

$$CLOD=(LOD-BOD)\times{BODs}/{BOD},$$

$$BODs=50$$

(CLOD, corrected leakage optical density; LOD, lesion optical density; BOD, background optical density; BODs, standard background optical density, Area, area of hyper-fluorescence (pixels); N, number of lesions in one laser treatment.)

The MIFI value was recorded for each eye at each time point and compares to the maximum MIFI value recorded for the particular eye. Statistics were calculated by two-way ANOVA test using Prism. A: Change of MIFI value after Laser 1. P<0.01 comparing Control group and Gel group at week 7 assuming the MIFI value is identical between week 7 and week 8. B: Change of MIFI value after Laser 2. P<0.05 comparing Bolus group and Gel group at week 10.
